# How Microbiomes Affect Skin Aging: The Updated Evidence and Current Perspectives

**DOI:** 10.3390/life12070936

**Published:** 2022-06-22

**Authors:** Yanisa Ratanapokasatit, Wannada Laisuan, Teerapong Rattananukrom, Aisawan Petchlorlian, Iyarit Thaipisuttikul, Mongkhon Sompornrattanaphan

**Affiliations:** 1 Division of Dermatology, Department of Medicine, Faculty of Medicine, Ramathibodi Hospital, Mahidol University, Bangkok 10400, Thailand; yanisa.rtn@gmail.com (Y.R.); teerpongrattananukrom@gmail.com (T.R.); 2 Division of Allergy Immunology and Rheumatology, Department of Medicine, Faculty of Medicine, Ramathibodi Hospital, Mahidol University, Bangkok 10400, Thailand; wannada.lai@mahidol.ac.th; 3 Division of Geriatric Medicine, Department of Medicine, Faculty of Medicine, Chulalongkorn University, Bangkok 10330, Thailand; aisawan.p@chula.ac.th; 4 Geriatric Excellence Center, King Chulalongkorn Memorial Hospital, The Thai Red Cross Society, Bangkok 10330, Thailand; 5 Department of Microbiology, Faculty of Medicine Siriraj Hospital, Mahidol University, Bangkok 10700, Thailand; iyarit.tha@mahidol.ac.th; 6 Division of Allergy and Clinical Immunology, Department of Medicine, Faculty of Medicine Siriraj Hospital, Mahidol University, Bangkok 10700, Thailand; 7 Faculty of Medicine Siriraj Hospital, Center of Research Excellence in Allergy and Immunology, Mahidol University, Bangkok 10700, Thailand

**Keywords:** aging, microbiota, microbiome, mycobiome, dermatological and cosmetological treatments, dietary, pharmacology, biology, probiotics

## Abstract

The skin has a multifactorial aging process, caused by both intrinsic and extrinsic factors. A major theory of aging involves cellular senescence or apoptosis resulting from oxidative damage as the skin’s antioxidant system tends to weaken with age. The human microbiota is a complex ecosystem that is made up of microorganisms (bacteria, fungi, and viruses). Both gut and skin microbiota have essential roles in the protection against invading pathogens, mediating inflammatory conditions, and the modulation of the immune system which is involved in both innate and adaptive immune responses. However, the human microbiome could be changed during the life stage and affected by various perturbations. An alteration of the intestinal bacteria results in “microbial dysbiosis” which is associated with the influence of various diseases, including aging. The skin interactome is a novel integration of the “genome-microbiome-exposome” that plays a significant role in skin aging and skin health. Mitigating the negative impacts of factors influencing the skin interactome should be the future strategy to protect, prevent, and delay skin aging along with preserving healthy skin conditions. This review summarizes the current evidence on how human microbiomes affect skin aging and demonstrates the possible interventions, relating to human microbiomes, to modulate skin health and aging. Probiotics-based products are currently available mainly for the add-on treatment of many dermatologic conditions. However, at this point, there are limited clinical studies on skin anti-aging purposes and more are required as this evolving concept is on the rise and might provide an insight into future therapeutic options.

## 1. Aging Process

Aging is the process of turning from a younger to an older, often less healthy, organism. While the age considered as ‘old’ or ‘aged’ is usually socially determined to be around 60 to 70 years old, the aging process itself occurs throughout life [1]. Biologic aging is a process occurring at the cellular and molecular level leading to a deterioration in the function and structure of all of the organ systems. Genetic factors play a major role in aging. Genetics determine the lifespan of a species and partly explain why individuals from some lineages tend to live a longer and healthier life than others [2]. Thus, it is generally accepted that aging is universal, unavoidable, and irreversible in our present scientific understanding. However, aging may be accelerated or delayed through the modification of extrinsic factors such as physical activity, dietary patterns, resting, exposure to environmental factors, and diseases. These factors impact the rate of aging through common molecular mechanisms that result in the accumulation of damage and diminished cellular recovery ability. The mechanisms that are currently believed to be the main drivers of aging are genetic and epigenetic alterations, the accumulation of abnormal proteins, oxidative stress and mitochondrial dysfunction, as well as cellular senescence [3].

**Figure 1:**
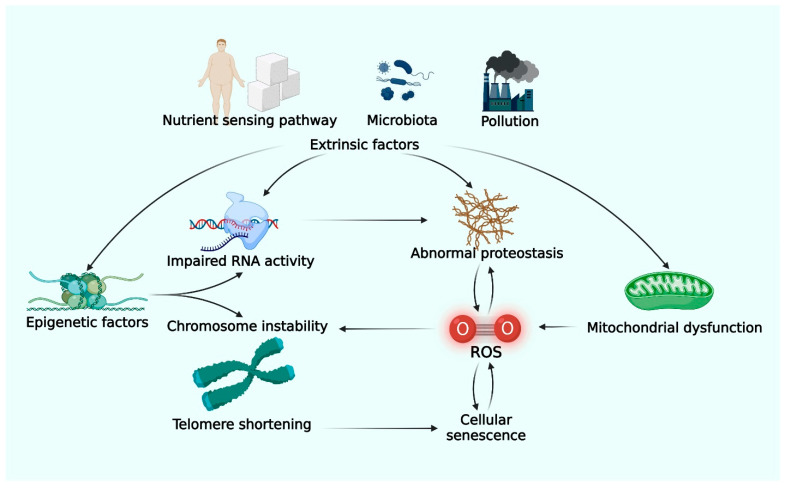
**Molecular mechanism of aging.** Intrinsic pathways believed to play a major role in aging are genetic and epigenetic alterations, accumulation of abnormal proteins, oxidative stress and mitochondrial dysfunction, and cellular senescence. Extrinsic factors such as pollutants, microbiota, and dietary nutrients also interact with intrinsic mechanisms and may accelerate or decelerate aging. (Created with BioRender.com) (accessed on 12 May 2022) Abbreviation: RNA; ribonucleic acid, ROS; reactive oxygen species.

DNA undergoes damage, mutation, and telomere shortening through exposure to oxidative stress and cell replication. Human cells have intrinsic DNA repair systems to maintain genetic stability. However, the repair system functionally declines over time leading to the accumulation of defects and cellular dysfunction [4]. Epigenetic phenomena, such as histone modification and DNA methylation, play roles in protecting the DNA and regulating gene expression [5]. Exposure to environmental pollution, ingestion of an unbalanced diet, or generation of toxic substances by microbiota can lead to abnormal alteration of epigenetic factors and eventually cellular dysfunction (Figure 1) [5,6].

The accumulation of abnormal proteins has been demonstrated as the main pathogenesis in various age-related degenerative diseases, such as dementia and Parkinson’s disease. Proteins may be abnormally synthesized from the pathologic genes or altered post-translation during phosphorylation, cleavage, folding, or packaging as a result of oxidative stress and impaired proteostasis system [7].

Reactive oxygen species (ROS) produced by mitochondria play deleterious roles in various processes of cell cycle regulation and immunity. Mitochondrial dysfunction produces excessive ROS and inflammation, which in turn damages DNA and synthesized proteins, leading to cellular aging [8]. Recent studies on aging have identified various pathways related to energy and nutrient metabolisms, such as insulin and the insulin-like growth factor-1 signaling pathway, the TOR pathway, and the action of sirtuin, which affects gene expression, protein modification, and regulation of mitochondrial function. These discoveries emphasize the impact of dietary patterns and caloric intake on aging [3].

Cellular senescence, defined as permanent cell cycle arrest, is a physiologic state of the cell and provides a way to suppress tumorigenesis. Cellular senescence would naturally occur with aging because human cells lack the telomerase enzyme. However, the aforementioned conditions of DNA damage and oxidative stress accelerate cellular senescence. Senescent cells are not only directly limited in their ability to regenerate to maintain organ function, but they also induce chronic inflammation of local tissue through the release of proinflammatory cytokines [9].

Recent studies using advanced sequencing and metagenomic tools have highlighted the association between the microbiome and many aspects of health. Microbiome composition dynamically changes throughout the human lifespan and has a bidirectional impact on health and illnesses [10]. A large cohort study found that a high proportion of *Bacteroides* and low biological diversity are associated with decreased survival in older adults [11].

## 2. Skin Aging

Given that it is the largest interface of our bodies, the skin has a multifactorial aging process, caused by both intrinsic and extrinsic factors [12]. The intrinsic factors, namely chronologic skin aging, seemingly entail a set of unavoidable physiologic changes in the skin that occur with time and are influenced by genetic, hormones, and cellular metabolic changes, including metabolites from the gut and skin microbiome [13]. These alterations demonstrate soft tissue changes including decreased collagen production, lower amounts of lipids, epidermal thinning, and the loss of subcutaneous fat. Intrinsically aged skin appears dry and pale with fine wrinkles and increased laxity [14]. Facial aging is characterized by an inverted triangle shape, which is caused by the combination of soft tissue changes, facial bone resorption, and recession. In contrast, the extrinsic factors of aging, mostly known as photoaging, include structural and functional changes caused by various environmental factors, the primary one being ultraviolet radiation (UV). Other exogenous factors consist of cigarette smoking, diet, chemical exposure, trauma, and air pollution. Extrinsically aged skin manifests deep wrinkles, laxity, coarseness, increased fragility, and multiple telangiectasias. In addition, photodamaged skin may exhibit depigmentation such as darkening and mottled pigmentation. The histological features include solar elastosis, reduced number of fibroblasts, and decreased amount of extracellular matrix [15].

A major theory of aging involves cellular senescence or apoptosis resulting from oxidative damage [16] as the skin’s antioxidant system tends to weaken with age [17]. The generation of reactive oxygen species secondary to normal aerobic metabolism, and exogenous factors, such as UV radiation, is the main cause of skin aging [18,19]. Oxidative damage leads to the upregulation of stress-related factors, such as hypoxia-inducible factors and nuclear factor-kappa B (NF-κB). These factors induce the expression of cytokines (interleukin (IL)-1, IL-6, vascular endothelial growth factor (VEGF), and tumor necrosis factor (TNF)-α), all of which are proinflammatory regulators of cell survival and modulators of matrix-degrading metalloproteins, leading to collagen degradation [20,21,22]. Moreover, oxidative stress also modifies telomere signaling. In general, the shortening of telomeres is the result of the inability of DNA polymerase to replicate the final base pairs of a chromosome secondary to serial cellular division. When the telomeres reach a “critically short” threshold, the cell undergoes proliferative senescence or apoptosis. Oxidative insult appears to provoke telomere shortening, which is a cause of skin aging [23].

## 3. Human Microbiomes

Table 1 summarizes the differences between human gastrointestinal tract (GIT) microbiota and skin microbiota communities. The human GIT houses a complex ecosystem that is made up of trillions of microorganisms such as bacteria, fungi, and viruses, referred to as the gut microbiota [24]. The gut microbiome works to maintain host health and homeostasis, mediate inflammatory conditions, and modulate the immune system through a delicate balance of commensal and pathogenic bacteria [25,26]. However, the gut microbiome may be changed depending on lifestyle, nutrition, bacterial infections, antibiotics, surgical interventions, frailty, and inflammation [27,28,29]. An alteration of the intestinal bacteria results in microbial dysbiosis. A state of dysbiosis is characterized by a reduction in bacterial species diversity and a decrease in beneficial bacteria. Microbial variation can potentially affect the function of the microbiome by increasing intestinal permeability while compromising the absorption of nutrition, food metabolization, and immune system regulation [24,30,31,32]. A disruption of the intestinal microflora and its associated consequences can influence the pathology of various diseases, including aging [33,34].

**Figure 2:**
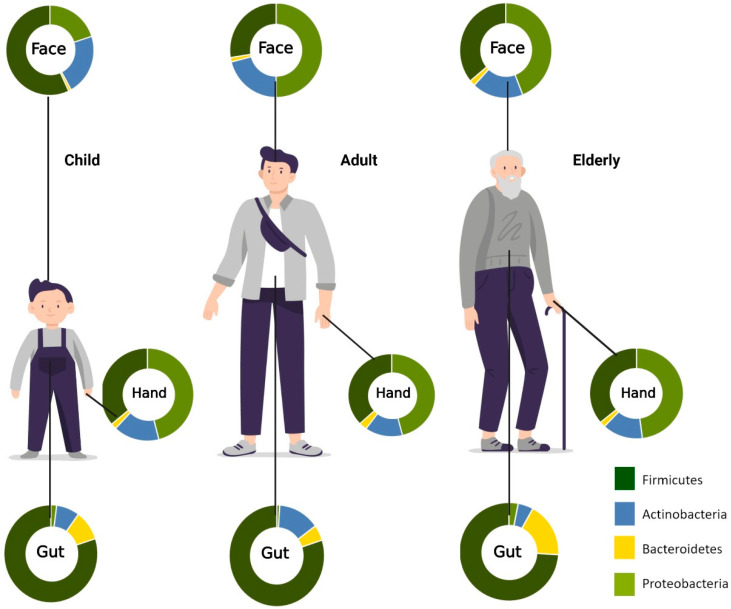
**Aging-associated changes in skin and gut microbiota composition.** Relative abundances of phyla on the face, hand, and gut. In the facial area, Firmicutes are most abundant in childhood, while Proteobacteria *is* most prominent in adulthood. For the hand microbiota, *Proteobacteria* was found to be the most predominant phylum from childhood to old age. *Firmicutes*, on the other hand, *are* most abundant in the gut across all age groups. (Created with BioRender.com) (accessed on 20 May 2022).

Skin is the largest organ in the human body. Similar to the gut microbiome, the skin microbiota is also composed of millions of microorganisms, including bacteria, fungi, and viruses. Some of these are beneficial symbiotics with essential roles in the protective barrier, preventing the invasion of pathogens. When an imbalance of commensals and pathogens occurs, skin disease or systemic disease can occur. Microorganisms reside at different depths or sub-compartments of the skin. Some microorganisms are variably present at the surface compared with deeper skin layers. Therefore, the capture of skin microbiota usually depends on the method used to sample these organisms. Most skin microbiome surveys have used amplicon sequencing. Recently, major technical and analytical breakthroughs have, however, enabled shotgun metagenomic sequencing studies. Additional studies with more invasive sampling techniques are necessary to fully understand the distribution of microorganisms in the skin. The overview of age-associated changes in gut and skin microbiota is demonstrated in Figure 2.

## 4. Skin Aging and Gut Microbiome

During the transition from adulthood to old age, the gut microbiota undergoes significant alterations. When compared to adults, there is a decline in microbial diversity and a greater inter-individual variation in microbiota composition in old people (>65 years old) [44]. It has also been demonstrated that microbiome composition can influence the rate of aging [29,45]. There is no known chronological threshold or age at which the microbiota composition abruptly changes; rather, these changes gradually occur over time [46]. The distinct microbial composition in the GIT has been attributed to aging and age-associated inflammation. For instance, a decline in the anti-inflammatory bacterial species was found in aged mice [47]. As shown in prior studies focusing on gut microbiota in centenarians, longevity is positively associated with an abundance of short-chain fatty acid (SCFA) producers, such as *Clostridium* cluster XIVa, Ruminococcaceae, *Akkermansia*, and Christensenellaceae [48,49]. The average phyla composition of centenarians was different from those of other elderly and adults. Furthermore, a recent study reported 116 microbial genes significantly correlated with aging, which were identified as a signature for longevity [50]. More diverse phyla were detected in the microbiota of centenarians compared to other groups. Additionally, good immunological and metabolic health-related bacteria, such as *Akkermansia*, Christensenellaceae, and *Lactobacillus*, were higher in centenarians than in other groups [51]. Correspondingly, the loss of *Lactobacillus* and *Faecalibacterium*, as well as the abundance of *Oscillibacter* and *Alistipes* genera along with the Eubacteriaceae family is linked to frailty in elderly people. Frail elderly people also have more proinflammatory *Bacteroidetes* commensals [44,52]. Controversially, some studies have shown centenarians’ microbiotas are less diverse than those of adult persons with decreased levels of *Bifidobacterium*, *Bacteroides*, and Enterobacteriaceae, and increased *Clostridium* spp. levels [48,53]. Several authors have suggested that the aging-associated differences in gut microbiota generally may not always be caused by aging, but they might be linked to a general decline in health status. According to age-related changes in microbiome diversity, recent findings have shown that a loss of diversity in the core microbiota groups is associated with aging-associated frailty rather than chronological age [44,46,54,55].

The production of a broad range of bioactive metabolites is a critical component of the gut’s function, serving as the most likely linkages between the gut microbiota and the host. SCFAs, such as butyrate, propionate, and acetate, are products of fiber fermentation by the gut microbiota and have been shown to exert anti-inflammatory and immunomodulatory effects [56,57,58,59]. A prior study demonstrated a dramatic decrease in the Firmicutes phyla and an increase in the Bacteriodetes phyla occurred from adulthood to old age, resulting in a decline in the Firmicutes-to-Bacteriodetes (F/B) ratio [60]. In particular, the F/B ratio is crucial for the production of SCFAs [61]. Generally, age-related dysbiosis can enhance the progression of aging, inflammation, and frailty, while compromising overall health and longevity.

Investigators have begun to explore the relationship between senescence and microbial dysbiosis. A recent study investigating the microbial composition in senescent models was performed [62]. In aged mice, the gut microbiome signatures associated with the markers of cellular senescence and inflammatory factors, known as senescence-associated secretory phenotype (SASP) were evaluated. Findings revealed that Clostridiales, *Staphylococcus*, and Lachnospiraceae positively correlated with all of the cellular senescence and inflammatory markers. Conversely, Coriobacteriaceae and *Akkermansia* correlated negatively with these markers. The relation between cellular senescence and microbial composition implies that microbial dysbiosis is involved in senescence. In addition, prebiotics and probiotics are efficient in preventing particular pathological conditions in elderly populations by suppressing inappropriate chronic inflammation and improving adaptive immune responses, thereby counteracting immunosenescence [63,64].

Furthermore, gut dysbiosis with age results in a leakage of proinflammatory microbial products via impaired intestinal permeability [52]. These products are then translocated into the bloodstream, leading to systemic effects. Microbial metabolites promote SASP damage through the upregulation of various inflammatory molecules, including tumor necrosis factor-alpha (TNF-α), interferon-gamma (IFN-γ), IL-1, IL-6, matrix metalloproteinases (MMPs), and others, contributing to the chronic proinflammatory state or inflammaging. As a consequence of dysbiosis, inflammaging and deficient immune surveillance thereby impair the removal of senescent cells.

## 5. Gut-Skin Axis and Skin Aging

**Figure 3:**
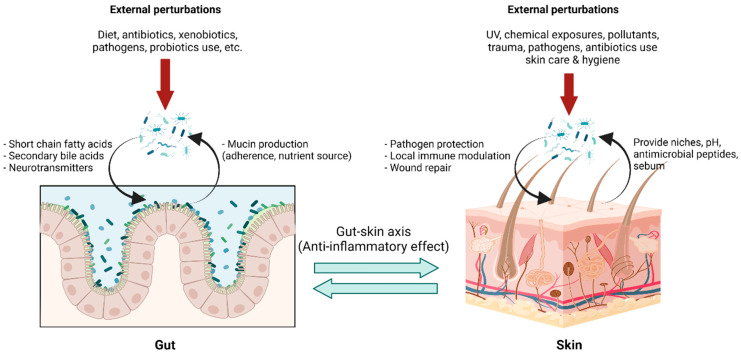
**Gut-skin axis in a homeostatic state:** The gut environment provides niches for gut microbiota with nutrients and optimal growth conditions, while gut microbiota carry out pleiotropic functions in maintaining body homeostasis. Microbial metabolites, for example, short-chain fatty acids, secondary bile acid, and several small molecules, not only locally maintain enterocyte functions but also exert systemic effects, including immune tolerance. This effect is linked to the skin via blood circulation, forming the so-called “gut-skin axis,” and provides an anti-inflammatory environment in the skin, optimizing interactions with skin microbiota. Skin microbiota in a homeostatic state help prevent pathogen colonization, modulating local immune responses and facilitating wound healing. Transient external perturbations, either cutaneous or mucosal, could disrupt microbiota composition, resulting in a transient dysbiosis state. The balanced microbiota could be recovered by perturbation removal, growth promotion of microbiota by proper diets, and, although in development, intervention with pro-and prebiotics. (Created with BioRender.com) (accessed on 12 May 2022). Abbreviation: pH, potential of hydrogen; UV, ultraviolet.

The gut-skin axis describes the bidirectional communication pathway between the gut microbiome and the integumentary system via its immunological and metabolic properties (Figure 3). Bacterial microbes and their metabolites that enter blood circulation can travel through the body and affect distant tissue organs and the skin [65,66]. Although it is difficult to ascertain a causal relationship between the gut microbiome and skin conditions, multiple studies indicate a link between them with several dermatological diseases being associated with gastrointestinal disorders and vice versa [67,68]. In addition, previous studies have demonstrated that the increased intestinal permeability caused by dysbiosis has led to an accumulation of bacterial metabolites in the skin, as well as impairment in epidermal differentiation and skin integrity [69,70]. The exact mechanism underlying gut-skin microbial interactions has not yet been fully elucidated. However, recent reports demonstrate oral probiotics to be beneficial in improving several signs of skin aging, including acidic skin pH, oxidative stress, photodamage, and skin barrier dysfunction [71]. Furthermore, studies determining the connection between *Lactobacillus plantarum* HY7714, *Bifidobacterium breve* B-3, and skin protection have been conducted. Findings suggest that there were functional substances in the skin–gut axis communication, which interact in a photoprotective manner, resulting in an anti-aging effect in a mouse model [72,73,74], and administration of *Lactobacillus plantarum* HY7714 can decrease the symptoms of UV-induced skin photo-aging in humans [75].

**Figure 4:**
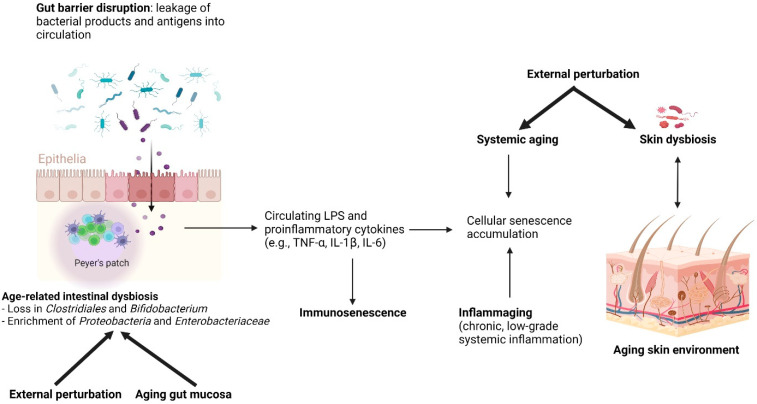
**Age-related intestinal dysbiosis:** Age-related intestinal dysbiosis is generally characterized by a decrease in short-chain fatty acid producers, for example, Clostridiales, and Bifidobacterium, and enrichment of pro-inflammatory *Proteobacteria* including the opportunistic Enterobacteriaceae. It is likely to be a result of aging gut mucosa and external factors, for example, drug use, diet, and behavioral changes. Gut dysbiosis leads to a state of “leaky gut,” described by increased permeability of the gut mucosa due to tight junction disruption, allowing a small but periodic translocation of bacterial contents into the systemic circulation. Bacterial antigen, especially lipopolysaccharide, is pro-inflammatory, increasing circulatory pro-inflammatory cytokines, for example, TNFα, IL-1β, and IL-6. Chronic exposures to pro-inflammatory bacterial antigens have been hypothesized to contribute to, in addition to the aging process, the accumulation of cellular senescence and immunosenescence, both of which lead to a state of chronic low-grade systemic inflammation called inflammaging. Inflammaging was thought to be the basis of age-related aberrant conditions, including the immune dysregulation of the skin, which consequently leads to skin microbiota dysbiosis. Skin dysbiosis is associated with several dermatological diseases, with a higher proportion of pathogen colonizers and pro-inflammatory microbiota. (Created with BioRender.com) (accessed on 18 April 2022). Abbreviation: IL, interleukin; LPS, lipopolysaccharide; TNF, tumor necrotic factor.

Gut dysbiosis (Figure 4), the impairment of senescent cell removal, and the accumulation of SASP factors can affect the function and integrity of the skin, leading to premature aging phenotypes. Notably, the upregulation of MMPs, which belong to SASP, is a contributory factor to age-related skin changes. MMPs reconstruct the extracellular matrix (ECM) by degrading proteins including collagen, fibronectin, elastin, and proteoglycans. The alterations made to the ECM by MMPs can influence skin wrinkling, sagging, and laxity [76,77]. However, the underlying mechanism addressing the relationship between the gut microbiome and skin aging characteristics has not yet been well established. Further studies are needed to advance the understanding of relationships between microbial composition, metabolite alteration, as well as accumulation, and skin phenotype changes.

## 6. Skin Aging and Skin Microbiomes

The skin microbiome plays a significant role in maintaining skin homeostasis and contributes to the skin’s barrier function to protect against the environment and potential pathogens [78]. Commensal bacteria compete for nutrients and space, inhibiting the reproduction of competitors via the production of antimicrobial compound peptides (AMPs), leading to inhibition against pathogen growth [78,79]. Skin microbes secrete enzymes involved in skin homeostasis; protease enzymes play a role in *stratum corneum* renewal, lipase enzyme is involved in lipidic film surface breakdown; and urease enzyme is implicated in urea degradation. Other roles of microbiota include the production of bacteriocin, quorum sensing, biofilms, and pH regulation by sebum and free fatty acid production [43]. In addition, the interaction between host tissue and microbiome resulted in the complex signals involved in innate and adaptive immune responses [80].

Age-related skin changes are attributed to combinations of internal factors (genetics and gender), environmental factors (pollution, sun exposure, and climate), and lifestyle factors (exercise, stress, sleep, nutrition, and skincare routine) [43,80,81]. Skin aging is characterized by a decrease in sebum, sweat, and immune function, resulting in significant alternations in the skin surface’s physiology, including lipid composition, sebum secretion, and pH. These affect skin dryness, collagen fragmentation, reduction in the total amount of collagen and elastin, as well as influencing the skin ecology, possibly shaping the skin microbiome [43,80]. Dimitriu, et al. studied bacterial microbiomes of 495 North American participants at four skin sites and the oral mucosa using 16s rRNA gene amplicon sequencing and found that demographics, lifestyle factors, physiology, and aging contribute to skin microbiota variations while the influence of ethnicity was the strongest association with the oral microbiomes [81].

Aging-related alteration of skin microbiome diversity has been described in several studies. Higher bacterial alpha diversity has been reported in older adults. A Japanese cohort study reported the difference in bacterial species between younger adults aged 21–37 years old and older adults aged 60–76 years old with skin site dependency. This study showed a significant increase in *Corynebacterium* on the cheeks and forehead and *Acinetobacter* on the scalp in the older group. In contrast, *Cutibacterium* decreased in the cheeks, forehead, and forearms [82]. A study in North America also found that aging is associated with an increased abundance of Corynbacterial taxa, including *C. kroppenstedtiin* and *C. amycolatum* in the forehead area [81]. Juge, et al. studied the changing of microbiota diversity in Western European women, revealing a higher alpha diversity on older skin than on younger. The taxonomic composition analysis showed a decrease in *Acinetobacter* and an increase in Proteobacteria on older skin. At the genus level, old-aged skin exhibited an increase in *Corynebacterium* and a decrease in *Cutibacterium* relative abundance [83]. In another study, Somboonna, et al. studied the skin microbiota in 30 healthy Thai females aged 19–57 years and found Firmicutes was the most abundant bacterium in healthy elderly adults and acne-prone young adults. In contrast, Gemmatimonadetes, Planctomycetes, and Nitrospirae are more prevalent in healthy teenagers [84]. Howard et al. investigated the skin microbiome in the Caucasian women aged 20–70 years, reporting an age-related decrease in the sebocyte gland area and an increase in the natural moisturizing factors (NMF), skin lipids, and antimicrobial peptides (AMPs), resulting in a decrease in the relative abundance of *Cutibacterium* and *Lactobacillus* at the face, forearms, and buttocks in the older age group [85].

Modifying skin physiology during the aging process, such as hydration, sebum secretion, pH, and lipid composition, could predict changes in microbiota. Mukherjee et al. studied the relationship between the facial skin microbiome and variations in sebum and hydration levels in healthy female volunteers and revealed an increase in cheek sebum increased the relative abundance of *Actinobacteria* and *Cutibacterium* whereas microbiome diversity decreased [86]. Moreover, cutaneous immunity is weakened with age, thus further impairing the skin barrier and increasing skin infections and cancer susceptibility. Skin aging altered the immune cell composition with reduced Langerhans cells decreased antigen-specific immunity and increased Foxp3+ regulatory T cells [80].

## 7. Possible Interventions to Modulate Skin Health and Aging

The skin interactome is a novel integration of the “genome-microbiome-exposome” that plays a significant role in skin aging and skin health [87].

Probiotics, particularly *Lactobacillus* and *Bifidobacterium*, are emerging as nutricosmetic agents to mitigate skin aging demonstrated by signs of aging skin including pH, oxidative stress, photodamage, and skin barrier dysfunction [71]. The *Bifidobacterium breve* strain YaKult can attenuate UV-induced barrier perturbation and oxidative stress of the skin in a mouse model [88]. *Lactobacillus plantarum* had the potential to prevent UV-induced photoaging by inhibiting MMP-1 expression in fibroblast in mice [89]. Lactic acid secreted by *Lactobacillus reuteri* DSM 17,938 can protect skin from UVB by suppressing pro-inflammatory IL-6 and IL-8 cytokines [90]. A randomized, double-blinded, placebo-controlled trial in 110 participants aged 41–59 years found that daily intake of *Lactobacillus plantarum* HY7714 for 12 weeks could significantly improve skin hydration, skin gloss, skin elasticity, and alleviate facial wrinkles compared with the placebo group [75]. In addition, Exopolysaccharide (EPS) produced by *Lactobacillus plantarum* HY7714 possess many biological activities, including immunomodulatory and antioxidant activity. HY7714 EPS regulates the intestinal tight junction in human intestinal adenocarcinoma cells (Caco-2) by upregulating the genes encoding occluding-1 (OCL-1) and zonula occluden-1 (ZO-1) that act on the gut-skin axis to change the properties of dermal cells [72]. Probiotics may work to restore the balance between free radical scavengers and free radical production.

A combination of probiotics and prebiotics can benefit skin conditions by increasing skin hydration and decreasing phenol production levels. A randomized, double-blinded, placebo-controlled trial in 600 healthy Japanese adult women found daily intake of *Bifidobacterium breve* strain Yakult and galacto-oligosaccharides (GOS) for four weeks could significantly improve skin hydration, increase cathepsin L-like activity (an indicator of keratinocyte differentiation), and decreased the serum and urine phenol in the active group [91].

Skincare focusing on improving skin health through formulations that contain prebiotics, probiotics, or skin microbiome-friendly ingredients is of growing interest. *Hylocereus undatus* fruit extract, a major source of antioxidants that may affect the balance of the skin microbiome, has been developed [92]. Topical formulations containing bacterial extracts have been investigated to understand their impact on the commensal skin flora. The tests specified included *Lactobacillus acidophilus*, *Lactobacillus plantarum*, *Lactobacillus reuteri*, a formulation based on a patented *Lactobacillus* mixture (CN110121353A), *Lactobacillus helveticus*, *Lactobacillus rhamnosus* synergistically applied with the plant *Agastache rugosa*, and *Bifidobacterium breve* [43]. Potential microbiota-targeted probiotic intervention in skin aging was summarized in Table 2.

Emerging evidence demonstrated that age-related epigenetic changes are also a potential target for future intervention [96,97]. Manipulation of epigenomic pathways might reverse epigenetic aberrations, which are a hallmark of aging [98]. Incorporating both microbiome analyses and genetic aging tests [99,100], some of which are commercially available now, might have potential roles for indicating the individual’s current status for clinicians and might direct clinicians’ decisions on how to intervene in physiologic aging. Similar to micro Biomed-targeting drugs, several drugs targeting epigenetic enzymes are now commercially available, and others are under clinical trial, but have not yet been studied in large confirmatory trials or long-term effectiveness studies [98].

## 8. Conclusions

**Figure 5:**
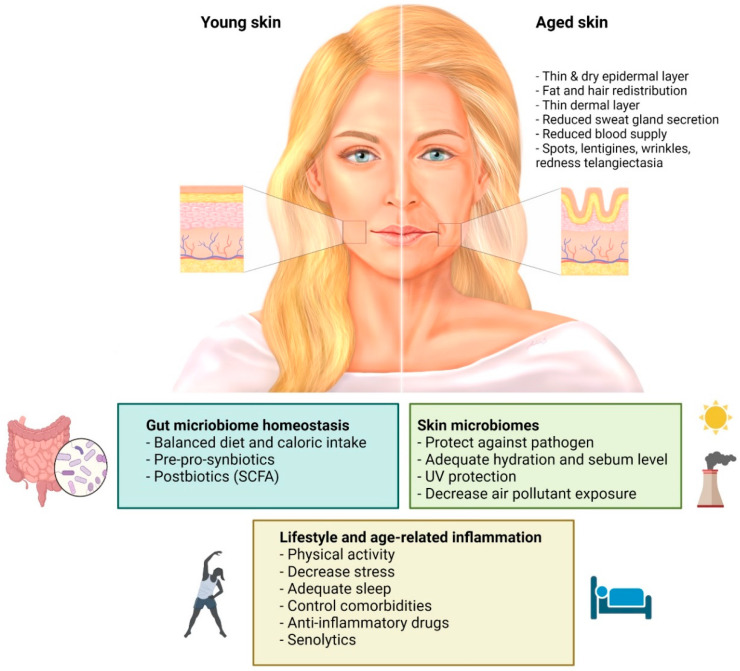
**Possible microbiome-targeted intervention to prevent skin aging:** Genome-microbiome-exposome play a significant role in skin aging and skin health. Age-related skin changes are attributed to combinations of internal factors, environmental factors, lifestyle factors, and skincare routines. The skin aging process results in changes in lipid composition, sebum secretion, and pH, affecting skin dryness, and collagen fragmentation, reducing the total amount of collagen and elastin, and influencing the skin microbiome. Modifying the factors that affect the skin aging process could be a possible intervention to improve skin health. Prebiotics and probiotics that act on the gut-skin axis possess many biological activities, including immunomodulatory and antioxidant activity, which can benefit skin conditions by increasing skin hydration, skin gloss, skin elasticity, and alleviating facial wrinkles. (Created with BioRender.com) (accessed on 12 May 2022). Abbreviation: SCFA, short-chain fatty acid; UV, ultraviolet radiation.

In conclusion, the skin interactome is a novel integration of the “genome-microbiome-exposome” that plays a significant role in skin aging and skin health. Mitigating the negative impacts of factors influencing the skin interactome should be the future strategy to protect, prevent, and delay skin aging along with preserving healthy skin conditions, as summarized in Figure 5. There are limited clinical studies on skin anti-aging purposes and more are required as this evolving concept is on the rise and might provide an insight into future therapeutic options. However, it is important to keep in mind that aging is a multi-factorial and multi-dimensional process. Maintaining an individual’s holistic health is still a very essential part of the current concept in medicine.

## Figures and Tables

**Table 1:** Differences between human gut microbiota and skin microbiota communities.

Comparators	Gut Microbiota	Skin Microbiota	Citation
Microbial biomass	Higher biomass, Lower diversity	Lower biomass, higher diversity	[35,36]
Initial colonization pattern	Affected by mode of delivery (i.e., normal labor or Cesarean section)	Acquired from close contact, for example, family members	[37,38]
Microbial distribution	Longitudinal variations; increases steadily along with GIT and a high number in the colon Horizontal variations; gut mucosa layers and gut lumen	Varies by skin physiological site (dry or oily)	[36,39]
Colonization stability	Stabilizes for at least 3–5 years and is likely to be maintained throughout life	Stabilizes for at least 2 years and significant changes during puberty due to hormonal influence	[36,40,41,42]
Microbiota community	Changed by antimicrobials and acquired GIT infection in later life	Changed by age, hormones, antimicrobial selection pressure, and environment	[36,39,43]

Abbreviation: GIT; gastrointestinal tract.

**Table 2:** Potential microbiota-targeted probiotic intervention in skin aging.

Author	Probiotics	Route	Study Design	Comparator	Result
Lee DE, et al., 2015 [72]	*Lactobacillus plantarum* HY7714: 2 g daily of a powder containing HY7714 (1 × 10^10^ CFU)	Oral	Randomized, double-blinded, placebo-controlled study (12 weeks)	Placebo	Increases in the skin water content in the face and hands Decrease trans-epidermal water loss At week 12, reduction in wrinkle depth, skin gloss, and skin elasticity
Kano M, et al. [91]	Milk containing GOS, polydextrose, *Bifidobacterium breve* strain Yakult (YIT 12272), *Lactococcus lactis* YIT 2027, and *Streptococcus thermophilus* YIT 2021	Oral	Randomized, double-blinded, placebo-controlled study (4 weeks)	Placebo	Prevented the decrease in hydration level in the stratum corneum Increased cathepsin L-like activity Decreased serum and urine phenol levels Decrease serum and urine phenol levels (*p* = 0.014, *p* = 0.002, respectively
Kimoto-Nira H, et al. [93]	*Lactococcus lactis* strain H61: 60 mg of lyophilized heat-killed *Lactococcus lactis* strain H61 cells in test food *	Oral	Double-blinded, placebo-controlled trial	Placebo	Maintained skin hydration Improved patient-self-reported skin elasticity
Ogawa M, et al. [94]	*Lactobacillus brevis* (*L. brevis*) SBC8803 (SBL88™), 25 mg and 50 mg heat-killed *L. brevis*	Oral	Randomized, double-blinded, placebo-controlled study (12 weeks)	Placebo	Improved skin hydrating conditions
Notay M, et al. [95]	*Nitrosomonas eutropha,* topical aerosolized live *Nitrosomonas eutropha* in buffer (AOBiome)	Topical	Prospective study (for 7 days)	High-dose versus low-dose	Higher reductions in wrinkles in the high concentration group

Abbreviation: CFU; colony-forming unit, g; grams, GOS; galacto-oligosaccharides, mg; milligrams. * Test food includes *L. lactis* subsp. *cremoris* H61, constituted broth (0.5% meat extract, 0.5% yeast extract, 1% sodium succinate, and 1% sodium chloride and 1% glucose) Alteration of physiology in skin aging, including lipid composition, sebum secretion, and pH, increases skin dryness and collagen fragmentation and reduces the total amount of collagen and elasticity, influencing the skin microbiome diversity [43,80,87]. Additionally, internal factors (genetics and gender), environmental factors (pollution, sun, and climate), and lifestyle factors (exercise, stress, sleep, nutrition, and skincare routine) also contribute to shaping the skin microbiota [43,81].

## Data Availability

Not applicable.

## Abbreviations

AMPs	antimicrobial compound peptides
CFU	colony forming unit
DNA	deoxyribonucleic acid
ECM	extracellular matrix
EPS	exopolysaccharide
F/B	Firmicutes-to-Bacteriodetes
g	grams
GIT	gastrointestinal tract
GOS	galacto-oligosaccharides
IFN-γ	interferon-gamma
IL	interleukin
LPS	lipopolysaccharide
mg	milligrams
MMPs	matrix metalloproteinases
NF-κB	nuclear factor kappa-light-chain-enhancer of activated B cells
NMF	natural moisturizing factors
OCL-1	occluding-1
pH	potential of hydrogen
RNA	ribonucleic acid
ROS	reactive oxygen species
rRNA	ribosomal ribonucleic acid
SASP	senescence-associated secretory phenotype
TNF	tumor necrosis factor
TNF-α	tumor necrosis factor-alpha
TOR	target of rapamycin
UV	ultraviolet radiation
UVB	ultraviolet radiation B
VEGF	vascular endothelial growth factor
ZO-1	zonula occluden-1
